# Maternal Nutrition during Pregnancy and Offspring Brain Development: Insights from Neuroimaging

**DOI:** 10.3390/nu16193337

**Published:** 2024-10-01

**Authors:** Xiaoxu Na, Philomena P. Mackean, Gracie A. Cape, Josiah W. Johnson, Xiawei Ou

**Affiliations:** 1 Department of Radiology, University of Arkansas for Medical Sciences, Little Rock, AR 72205, USA; 2 College of Medicine, University of Arkansas for Medical Sciences, Little Rock, AR 72205, USA; 3 Department of Pediatrics, University of Arkansas for Medical Sciences, Little Rock, AR 72205, USA; 4 Arkansas Children’s Research Institute, Little Rock, AR 72202, USA; 5 Arkansas Children’s Nutrition Center, Little Rock, AR 72202, USA

**Keywords:** maternal nutrition, pregnancy, offspring brain development, neuroimaging

## Abstract

Maternal nutrition during pregnancy is known to be important for offspring growth and health and has also been increasingly recognized for shaping offspring brain development. On the other hand, recent advancements in brain imaging technology have provided unprecedented insights into fetal, neonatal, and pediatric brain morphometry and function. This review synthesizes the current literature regarding the impact of maternal nutrition on offspring brain development, with a specific focus on findings from neuroimaging studies. The diverse effects of maternal nutrients intake or status during pregnancy on neurodevelopmental outcomes in children are discussed. Neuroimaging evidence showed associations between maternal nutrition such as food categories, macronutrients, and micronutrients including vitamins and minerals during pregnancy and child brain imaging features measured using imaging techniques such as ultrasound, magnetic resonance imaging (MRI), electroencephalography (EEG), and magnetoencephalography (MEG). This review demonstrates the capability of neuroimaging in characterizing how maternal nutrition during pregnancy impacts structure and function of the developing brain that may further influence long-term neuropsychological, cognitive, and behavioral outcomes in children. It aims to inspire future research utilizing neuroimaging to deepen our understanding of the critical impacts of maternal nutrition during pregnancy on offspring brain development.

## 1. Introduction

Human brain development starts as early as the third week of gestation and lasts into young adulthood. Gene expression and environmental input interact to support the ongoing series of events that contribute to brain development [1], which is a complex, dynamic, and adaptive process that supports both brain maturation and functioning. With recent advances in brain imaging technology, the capability of investigating fetal, neonatal, and children’s brain morphometry and function has opened a new avenue into understanding the developing brain. Brain imaging studies have provided solid insight into the complex trajectories of brain structure and functional development from fetus to childhood [2,3].

A number of recent studies have suggested, through neuroimaging techniques, that maternal nutrition intake or status during pregnancy is associated with differences in offspring brain development. The purpose of this review is to summarize and discuss relevant findings in the literature to intrigue more neuroimaging studies that investigate relationships between maternal nutrition and offspring brain development. This review will start by briefly summarizing findings of how maternal nutrition potentially impacts neurodevelopmental outcomes, not limited to neuroimaging studies. It is suspected that direct impacts on the developing brain may be the mediating factor between maternal nutrition during pregnancy and offspring’s long-term neuropsychological, cognitive, and behavioral outcomes [4,5,6]. Different maternal nutrients and their potential impacts on offspring brain development reflected by neuroimaging will then be discussed in detail. Finally, challenges and limitations in published studies and future clinical studies in terms of determining a causal relationship of maternal diet during pregnancy on offspring neurodevelopment are briefly discussed.

## 2. Maternal Nutrition during Pregnancy and Offspring’s Neurodevelopmental Outcomes

A solid nutritional foundation during pregnancy is vital for ensuring a healthy pregnancy and positive long-term outcomes for both the mother and the child. The latest Dietary Guidelines for Americans (DGA) 2020–2025 has a chapter specifically aimed for women who are pregnant or lactating [7], with a brief summary of recommendations for food and beverages during pregnancy listed in Table 1. In general, the core elements of a healthy diet for pregnant women include vegetables, fruits, whole grains, seafood, eggs, beans, peas, lentils, unsalted nuts and seeds, fat-free and low-fat dairy products, lean meats, and poultry. In addition, DGA 2020–2025 strongly advocates for seafood consumption due to its association with favorable measures of cognitive development in young children. It is possible for maternal diets during pregnancy to be insufficient in providing the essential nutrients for proper fetal growth [8,9], and therefore dietary supplements are often recommended during pregnancy to fill the gap. Specifically, DGA 2020–2025 highlighted some vitamin and mineral guidelines for pregnant women; for example, all women should take a daily supplement containing 400 to 800 mcg of folate in addition to the amounts of food folate contained in a healthy eating pattern at least 1 month before conception and continuing through the first 2 to 3 months of pregnancy. Iron, iodine, choline, and other supplementations are also recommended due to the increased need for them during pregnancy.

Both deficiencies and excesses of key nutrients during pregnancy may lead to altered fetal programming and contribute to unfavorable future outcomes [5]. For example, a meta-analysis review showed that maternal iron deficiency during pregnancy was associated with adverse outcomes for infants and children in cognitive, memory, motor, and language functioning, while maternal iron excess during pregnancy showed lower cognitive scores in infants [10]. Additionally, reviews also suggested that maternal folate deficiency was associated with smaller total brain volume, altered cortical thickness, and changes in cerebral white matter in children [4], while maternal over-supplementation of folate may increase the risk of autism spectrum disorder (ASD) in children [11].

Overall, published scoping reviews have highlighted the importance of many essential nutrients, including vital macronutrients and micronutrients that play an important role in successful fetal neurodevelopment [6,12,13] and the potential prevention of adverse neurodevelopmental events [12]. An unbalanced diet and nutrition intake/status during pregnancy may increase the risk of offspring developing neurodevelopmental or neuropsychiatric disorders (e.g., ASD, attention-deficit/hyperactivity disorder, schizophrenia, anxiety, depression) [6,14,15], behavioral disorders [6,15], altered cognition (e.g., learning difficulties, decreased IQ, poorer language and memory development) [6,16,17], and/or visual impairment and motor deficits [6] due to potential genetic re-programming, neuroinflammation, and gut microbiome-mediated endotoxicity [15]. Notably, some neurodevelopmental outcomes, such as executive function which is one of the last cognitive domains to develop, would not be measurable in fetuses and neonates. Therefore, it is highly valuable to use advanced technologies to study the effects of maternal nutrition on brain development which may predict long-term neurodevelopment, before the outcomes actually manifest and become measurable.

## 3. Maternal Nutrition during Pregnancy and Offspring’s Brain Development Reflected by Neuroimaging

Neuroimaging is an effective technique to assess human brain structure and function, providing vital insights through a non-invasive and sensitive approach. Magnetic resonance imaging (MRI), positron emission tomography (PET), computed tomography (CT), and ultrasound are the most popular clinical techniques for brain structural imaging. They offer the opportunity to visualize and evaluate brain macrostructural and microstructural development [18,19,20,21]. MRI has no radiation and therefore is more feasible for research compared to PET and CT. Additionally, functional imaging modalities such as functional MRI, electroencephalography (EEG), and magnetoencephalography (MEG) can measure brain activation [22,23]. Furthermore, neuroimaging research of the developing brain at early ages, while historically challenging in the past, has become more widely feasible due to better sensitivity of imaging equipment, shorter imaging data acquisition time, and more sophisticated imaging post-processing tools. Together, these advances in neuroimaging technology have revolutionized our understanding of human brain development by expanding basic neurobiology and integrating it with psychiatry, psychology, neurology, and related therapeutic development.

This review includes studies investigating the associations between human maternal nutrition during pregnancy and offspring’s brain development assessed by neuroimaging (Table 2). PubMed and ScienceDirect were the electronic databases used for searching the literature published up to 31 August 2023. The search strategies were limited to only English-language publications and included terms related to both maternal nutrition during pregnancy and offspring neuroimaging assessments. Specifically, the strategies involved a combination of one nutrition keyword AND one neuroimaging keyword AND any terms reflecting pregnancy AND “child(ren)” OR “infant” OR “neonate”. Studies exploring maternal nutrients (macronutrients, micronutrients, etc.) were included, not limited to the assessment of a singular nutrient or food element but also food groups. Studies evaluating maternal nutrition during pregnancy at multiple levels, including nutrient intake, food intake, and nutrient status were included. Only studies which examined maternal nutrition during pregnancy were included. Studies with offspring’s brain development assessed by structural or functional measures using neuroimaging technologies, such as ultrasound, MRI, EEG, and MEG were included. Those only focused on psychological or behavioral outcomes without neuroimaging assessment were not included. Only studies on human beings were included. Only original articles and proceedings were included. All different types of study designs (e.g., prospective, retrospective, longitudinal, cross-sectional, randomized controlled trial, other trials) were included. In total, 31 studies met the inclusion/exclusion criteria and are presented in Table 2. With reference to U.S. 2020 Dietary Guidelines Advisory Committee’s Scientific Report [24], maternal nutrition was reviewed at different levels/angles including food categories, macronutrients, and micronutrients for the generation of Table 2.

### 3.1. Food Categories

As aforementioned, a healthy diet for pregnant women includes a combination of various foods and beverages. There are some studies that had reported specific intake of foods or beverages during pregnancy that showed an association with offspring’s brain development. For example, frequency of seafood consumption in the mid to late pregnancy was positively associated with fetal biparietal diameter (BPD) [26] and pattern-reversal visual evoked potentials at 2 years old [27], indicating better neurodevelopment overall or within the visual system during infancy. Another study found that fetal HC was also positively associated with a pattern of high proportion of more animal internal organs, thallophyte, and shellfish intake in the second trimester and more meat/fewer nuts intake in the third trimester, respectively, potentially due to the benefits of those foods that are rich in micronutrients, cholesterol and animal protein [25]. On the other hand, the study also found fetal HC was negatively associated with a pattern of high proportion of vegetables/fish intake and more snacks/fewer eggs consumed during the second trimester, respectively. The authors speculated that vegetables were fiber-rich food that might result in increasing post-meal satiety and reducing food intake, while fish were not only rich in fatty acids, but on the other hand can also be a source of pollutants such as methylmercury.

Ultra-processed foods (using the NOVA classification) are not encouraged and should be limited to daily intake, since these groups of foods are industrial formulations made entirely or mostly from substances extracted from foods, derived from food constituents, or synthesized in laboratories from food substrates. They are therefore of high energy density and low in fiber, protein, and necessary micronutrients, and have a potential impact on the brain reward system [56]. Nevertheless, consumption of ultra-processed food increased in many countries worldwide [57,58,59]. Higher ultra-processed food intake was associated with cognitive deficits such as IQ and verbal comprehension for children aged 4–7 [60], and cognitive impairment and higher risks of stroke for adults [61]. In neuroimaging studies, fetal HC measured in late pregnancy using ultrasound was decreased in those with more maternal consumption of ultra-processed food during the third trimester [28] and higher frequency of soda consumption [26].

It is noteworthy that many of the aforementioned neuroimaging studies only assessed fetal HC since it is convenient to extract from medical records. Also, some studies had limited sample size. Despite these limitations, all studies consistently showed that maternal food/beverages intake during mid to late pregnancy in accordance with DGA recommendation resulted in better offspring brain developmental outcomes. Further studies with larger sample sizes, diverse populations, more advanced neuroimaging features assessment, and longitudinal designs will be important to gain a more comprehensive understanding of the role of food category or pattern on the developing brain.

### 3.2. Macronutrients

Macronutrients are those needed in large amounts by the human body to function properly. These include carbohydrates, fats, and proteins. They are all highly involved with brain structural emergence and functioning, and maternal intake of macronutrients transferred via placenta provides the necessary supply to fetal brain development.

#### 3.2.1. Fatty Acids

Dietary fatty acids are extensively involved in essential processes in the human brain. The brain is highly enriched in lipids, which play a vital role in neurotransmission, synaptic plasticity, and inflammation, and fatty acids are directly involved in the structure of most lipids and therefore are crucial for brain function. These fundamental roles of fatty acids as structural components and functional modulators, including their involvement in maternal metabolism, are essential for feto-placental development [62]. Fatty acids can be divided into four general categories depending on their biochemical structure: saturated fatty acids (SFAs), *trans* fatty acids (TFAs), monounsaturated fatty acids (MUFAs), and polyunsaturated fatty acids (PUFAs). The relationships observed between each category and brain development are described in the paragraphs below.

SFA increase has recently been found to be implicated in the prevalence of brain diseases, particularly mood disorders [63]. However, SFAs (particularly saturated free fatty acids such as myristic and palmitic acids) were also suggested to have a critical role in memory acquisition [64]. Different maternal SFAs may result in opposite impacts on fetal/neonatal brain development. Specifically, higher maternal plasma phospholipid odd-chain SFAs in early pregnancy were positively associated with fetal HC and BPD [30]. On the contrary, maternal SFA portion in serum phospholipid fatty acids at the third trimester showed negative correlation with 2-year-old children’s pattern-reversal visual evoked potentials assessed by EEG [27]. Maternal saturated free fatty acids measured in fasted blood samples during pregnancy were inversely associated with neonatal brain microstructural integrity, which was also associated with early childhood body fat [29]. Additionally, higher maternal plasma phospholipid even-chain and very long even-chain SFAs were inversely associated with fetal HC and BPD at early pregnancy [30]. Furthermore, Li et al.’s study also suggested the relevance of timing in fetal HC and BPD being associated with maternal SFAs started in early–mid pregnancy (10–15 weeks of gestation) but attenuated in mid–late pregnancy (23–40 weeks of gestation).

Dietary TFAs come from natural food products from ruminant animals and a reducing quantity from hydrogenated oils formed during the manufacturing process. TFAs are already known to cause adverse cardiometabolic conditions [65]; however, little is known about the potential impact of maternal TFAs during pregnancy on offspring’s brain development. A recent ultrasound study revealed an inverse relationship between maternal plasma TFA concentration during mid-pregnancy and fetal HC in the third trimester and the growth of fetal HC from the second to the third trimester, respectively [31]. However, the association was unclear at age 10 years follow-up assessed by MRI.

Unlike SFAs and TFAs, many PUFAs are well-known and considered as essential fatty acids for the brain [66,67]. Researchers have investigated the relationships of different maternal PUFA statuses during pregnancy with child brain morphology. PUFAs are further divided into two families, omega-3 and omega-6. Maternal omega-3 PUFAs (including long-chain and short-chain) concentration during mid-pregnancy positively correlated with children’s total gray matter/white matter volume measured at 9–11 years of age by MRI [32] and pattern-reversal visual evoked potentials assessed by EEG at 2 years old that indicated better neurodevelopment within the visual system during infancy [27]. In addition, omega-3 PUFAs supplementation, such as docosahexaenoic acid (DHA) and eicosapentaenoic acid (EPA), together with arachidonic acid (ArA), were found to be associated with significantly larger total brain volume, total gray matter volume, and corpus callosum and cortical volumes in male neonates [33]. Furthermore, it was found that omega-3 PUFAs supplementation was associated with weaker brain functional connectivity of children at age 10 years in the default mode, sensorimotor, and frontal-parietal networks, indicating greater cognitive neurodevelopment and a tendency toward better memory [36], but not with attention network evaluation at age 8.5 years, as measured by EEG/event-related potential (ERP) [34], or total brain volume assessed at age 10 years [35]. Maternal omega-6 long-chain PUFA status during pregnancy was unrelated to the children’s gray matter or white matter microstructure at the age of 9–11 [32]; on the other hand, ArA coupled with DHA and EPA during pregnancy was associated with significantly larger total brain volume, total gray matter volume, and corpus callosum and cortical volumes in male neonates [33]. Since in the Ogundipe et al. trial the main group was supplied with both omega-3 and omega-6 fatty acids, it is impossible to assess their impact separately.

Finally, the influence of maternal MUFAs on the developing brain remains to be explored, although the concentration of MUFAs was found to promote the dorsal attentional network [68] and slow down cognitive decline in elderly adults [69]. The only neuroimaging study available is one with a small dataset (n < 20) in which maternal MUFAs in serum phospholipid fatty acids at the third trimester of pregnancy showed no correlation with pattern-reversal visual evoked potentials assessed by EEG at 2 years old [27].

#### 3.2.2. Carbohydrates

Glucose is the primary sugar that the body breaks down from carbohydrates to use as energy, and it appears to be the major substrate for placental and fetal energy metabolism that is dependent on additive effects of fetal plasma glucose and insulin concentrations [70]. Most of the energy produced from glucose metabolism is used for neuronal signal transmission functions such as synaptic transmission and non-signal activities like axonal transport and cytoskeleton remodeling [71]. Research on maternal carbohydrates intake during pregnancy and offspring brain development is scarce, although its association with child growth has been widely studied. So far, there have only been one study focusing on brain development, which revealed the interlink between maternal total and added sugar intake in pregnancy and early brain tissue organization in infants [37]. Specifically, maternal total and added sugar consumption during the second trimester were associated with inverse changes in mean/radial/axial diffusivity values in the neonatal brain measured by diffusion tensor imaging (an MRI method) throughout the cortical mantle, including the posterior periphery and frontal lobe.

#### 3.2.3. Protein

Protein also affects neurotransmitter function since the central nervous system (CNS) requires a number of amino acids found in protein foods. Amino acid transporter activity depends on the total concentration of amino acids available for transport from maternal side and on their relative concentrations compared with placenta [72]. Taken from the placenta, amino acids such as tryptophan, tyrosine, histidine, and arginine [73] are used by the brain for the synthesis of various neurotransmitters and neuromodulators [74]. So far, there is only one study reported findings in relationships of maternal protein intake and offspring brain development. Better outcomes of fetal HC and BPD assessed by ultrasound were associated with maternal protein consumption of 50–70 g/day rather than <50 g/day or >70 g/day measured in the third trimester [38].

### 3.3. Micronutrients

#### 3.3.1. Vitamins

##### Vitamin B

In addition to macronutrient components, it is important that pregnant women consume adequate but not excessive micronutrients such as vitamins to support their offspring’s cognitive development and functioning [75]. For example, folate is an established vitamin that helps form the neural tube and prevents major congenital abnormalities in the baby’s brain and spine. Humans cannot synthesize folate, and the requirement for folate in pregnant women is 5–10-fold higher than that of non-pregnant women. Therefore, adequate folate intake is essential for pregnant women. Children exposed to lower maternal folate concentration assessed via venous blood in early gestation had persistently smaller total brain volume in early childhood [39,40], smaller cerebral white matter volume at 9–11 years old [39], lower cortical thickness in the bilateral frontal and temporal region, and delayed cortical thinning in temporal and parietal regions at age 8–18 years [41]. When examining the impact on children’s brain functioning, an EEG study revealed that folate supplementation from the 20th week of pregnancy until delivery had improved 8.5-year-old children’s ability to solve response conflicts based on higher activation of the mid-cingulate cortex [34]. An MEG assessment of neuronal responses to a language task showed increased power at the Beta and High Gamma bands in 11-year-old children from mothers with folate supplementation throughout the whole pregnancy compared with supplementation intake only at the first trimester, suggesting more efficient semantic processing of language [43].

When mothers were exposed to low vitamin B12 at mid-pregnancy simultaneously with higher folate, less cortical thickness in multiple temporal and parietal areas was observed in young adults [42]. In addition, maternal vitamin B12 assessed in the second trimester was also found to have an interaction with the folate status and its influence on young adults’ subcortical volumes [42]. Specifically, higher maternal folate was associated with greater subcortical volume in the high vitamin B12 group but lower volumes in the low vitamin B12 group. However, vitamin B12 supplementation alone during pregnancy did not make a difference in children assessed for attentional resource allocation and brain efficiency measures using ERP amplitudes and latencies at age 6 years [44]. At the same time, with intervention group and placebo group combined, higher third-trimester maternal methylmalonic acid concentration was associated with lower amplitude in children, indicating late pregnancy maternal vitamin B12 status positively impacts attentional resource allocation in children.

##### Choline

Choline is also essential during fetal neurodevelopment due to its impact on cell proliferation and apoptosis [76]. EEG studies disclosed that greater choline levels in mothers during the second trimester were associated with improved newborn/infancy inhibition of auditory cerebral response [45,46,47]. Among these, one study also pointed out that a higher maternal choline level may improve the side effects of maternal infection on offspring neurodevelopment [46]. Another study found that in the presence of gene mutations that may delay infant inhibition, choline-treated infants aged ~1 month showed better outcomes as reflected by P50 inhibition suppression ratio [47].

##### Vitamin D

Vitamin D is a potent neurosteroid that mediates numerous actions in the brain, and the impact of maternal vitamin D and its metabolites on fetal brain development warrants further investigation [77]. Children exposed to persistently deficient vitamin D from mid-gestation to delivery showed less cerebral gray matter/white matter volumes, less gyrification, and smaller surface areas on MRI measurements at age 10 years [48].

As a summary for vitamins, it is quite promising that maternal vitamin B-group either assessed by status or oral intake from supplementation facilitates offspring brain development and functioning. This effect was also observed from early childhood to early adulthood, suggesting a persistent impact on neurodevelopment. Additionally, the duration and dose of vitamin B group supplementation during pregnancy have also been explored in the intervention trial [44] and the follow-up from another trial [43]. From a neuroimaging perspective, choline and vitamin D would also benefit children’s brain development, while the impacts of other vitamins remain unexplored.

#### 3.3.2. Minerals

##### Iron

Minerals (oligo-elements or trace elements), in addition to some vitamins, cannot be synthesized by the human organism. Thus, they must be acquired either through food or by supplementation when necessary. Iron is known to be vital during fetal neurodevelopment, as it impacts intracellular metabolism and monoamine production, which in turn affects socio-emotional development, executive function, and memory processes [78]. There is growing evidence indicating that there is a reshaping of the offspring’s brain associated with maternal iron intake during pregnancy. The demand for iron escalates during pregnancy to support fetal-placental development. The severity of iron deficiency anemia during the third trimester was reported to be inversely associated with neonatal hippocampal volumes [49]. Meanwhile, human pregnancy adjusts this demand starting in the second trimester by suppressing iron storage to facilitate an increased supply into the circulation [79]. On the other hand, excessive maternal iron intake during pregnancy was also associated with lower neonatal fractional anisotropy (FA) values predominately in cortical gray matter as assessed by diffusion tensor-measured MRI [50], and reduced brain growth assessed by structural MRI at the age 8–12 years [51] and fetal BPD via ultrasonography [52].

##### Iodine

Iodine is another vital mineral for fetal neurodevelopment since it is essential for the synthesis of thyroid hormones that, in turn, are required for brain development [80]. Like iron, maternal iodine excretion showed a curvilinear correlation with children’s brain morphology measured at 10 years old by MRI, suggesting both low and high maternal urinary iodine concentration would be associated with smaller offspring gray matter volume [53].

##### Zinc

Zinc also has a critical impact on fetal growth during pregnancy [81] as it is involved with cellular integrity and many biological functions including protein synthesis and nucleic acid metabolism, while the understanding of its direct effect on brain development remains limited. A recent MRI study found maternal zinc intake from diets was inversely associated with newborn FA migration toward the midbrain and mean diffusivity in the peripheral regions, but positively related to infant resting cerebral blood flow toward the hindbrain [54].

##### Sodium

High sodium intake is well-documented as a risk factor for high blood pressure, heart attack, and stroke; however, its contribution to brain functioning remains unclear. There is some evidence that high salt intake is associated with poor cognition in late adulthood [82]; on the contrary, children born prematurely and supplemented with more sodium intake in the first two weeks of postnatal life showed improved neurodevelopment at 10–13 years old [83]. A new study revealed that maternal sodium intake during the first trimester of pregnancy was negatively associated with neonatal white matter development, involving the anterior corona radiate, parietal white matter, posterior limb of internal capsule, external capsule, and temporal white matter as assessed by MRI [55].

As a summary for minerals, sufficient maternal minerals intake is important for offspring neurodevelopment. Findings from neuroimaging suggested both excess and deficiency of either iron or iodine may negatively impact the child’s brain. This effect of iron is consistent among studies of fetuses [52], neonates [50], and school-aged children [83], while the impacts of maternal iodine were only evaluated at 10 years old [53] and the impacts of maternal zinc and sodium intake were only evaluated in neonates [54,55].

## 4. Limitations and Future Research

Assessment of daily nutrient intake is complex even though there are different tools to assess it, such as various food frequency questionnaires, food records, and many customized tools. In our reviewed studies, maternal dietary intake was assessed by 3-day food records in two studies [27,55], by 24 h food questionnaire in six studies [26,37,38,50,52,54], and by a semi-quantitative food frequency questionnaire [84] in the Qin et al. study [25]. The 24 h food recall survey includes dietary intake records of both variety and portion size. However, it was suggested to combine this with an additional food frequency questionnaire to improve the accuracy of estimates of individual intakes [85]. Some studies reported the frequency of food taken or stated using a simplified food questionnaire [28,38], but not enough details were given. On the other hand, using a 3-day food record is sometimes desired, which is regarded as a more reliable measure of diet [86]. Furthermore, nutrition status assessed from blood samples may be confounded by experimental design. For example, fatty acids may be affected due to fasting status [87]. Some of the studies in the review [27,29,32,45,51] described fasting status for blood collection, while others did not.

In addition, different nutrients may have potential interacting effects on brain development implied from preclinical studies. For example, maternal vitamin B12 supplementation increased brain BDNF (protein and mRNA) and DHA levels in pups at birth and the effects were further enhanced by additional fatty acid supplementation [88]. Additionally, there are other nutrients such as creatine that may interrupt oxidative stress or fetoplacental hypoxia [89], which are also possibly involved in brain development, but relationships between their intake during pregnancy and offspring brain development remain unexplored. Most existing works in the literature of human research have a specific interest in one or two pre-selected nutrients, while studies on the entire nutrition pattern are challenging and rare.

**Figure 1:**
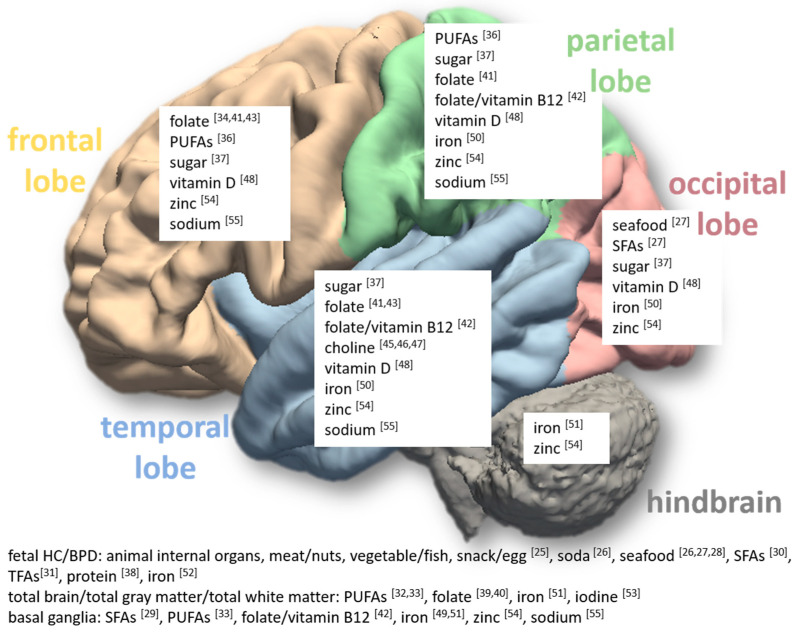
Different maternal nutrition components during pregnancy have potential impacts on various regions in the offspring brain, including frontal lobe, parietal lobe, occipital lobe, temporal lobe, hindbrain, basal ganglia, and the whole brain. The numbers in the figure refer to the referenced articles.

Furthermore, although it is clear that adequate maternal nutrition is important for brain development, different brain regions may be affected by various maternal nutrition components during pregnancy (as illustrated in summary in Figure 1). The time window of the impact on offspring brain development is inconclusive. In our review, the impact can be detected as early as during fetus and neonatal stages, and can also be detected in late childhood and early adulthood. The human brain undergoes significant changes over this long time span, with the normal brain trajectory following a sex-specific, asymmetric, and potentially ethnicity-specific pattern [90,91]. This complex development is also affected by many other factors during pregnancy and after birth. In our review, only a few studies [25,31,46,47] had more than one neuroimaging assessment to evaluate the persistency of the impacts associated with maternal nutrition. Many covariates and potential confounders will need to be considered to establish a causal relationship between differences in maternal nutrition and changes in offspring brain development.

## 5. Conclusions

Maternal nutrition during pregnancy is crucial for fetal brain development and may have prolonged impacts on cognitive, behavioral, and psychological outcomes in children. Either lack of or overabundance of vital nutrients may influence early brain development due to its volatility. There is no doubt that a well-balanced and nutrition-rich diet is essential during pregnancy, although the underlying mechanisms of how maternal nutrients affect brain development are still unclear.

With drastic improvements in technical tools to post-process and analyze neuroimaging data, traditional challenges in developmental neuroimaging, such as motion artifacts and low imaging contrasts between different tissue types, have been addressed. These improvements have been significant for the earliest imaging of the developing brain. Brain imaging templates appropriate to different age groups have refined the accuracy of imaging registration and highlighted the trajectory of the developing brain. The ability to detect subtle deviation from normal development before any potential psychiatric or behavioral symptoms emerge has become feasible. We expect pediatric neuroimaging to play an increasingly pivotal role in elucidating how maternal nutrition impacts offspring brain development in humans. Future studies should also focus on longitudinal studies of pediatric brain development to evaluate the persistence of effects associated with maternal nutrition during pregnancy and the impacts on long-term neurodevelopmental outcomes.

## Figures and Tables

**Table 1:** Food and beverage recommendations during pregnancy.

Foods and beverages encouraged	Vegetables, fruits, whole grains, eggs, beans, peas, lentils, unsalted nuts and seeds, fat-free and low-fat dairy products, seafood with low methylmercury, lean meats, and poultry when prepared with no or little added sugars/saturated fat/sodium
Food and beverages to be avoided	Seafood such as shark, swordfish, and king mackerel; beverages with alcohol, unpasteurized juice or milk, raw sprouts, or certain soft cheeses made from unpasteurized milk

**Table 2:** Overview of human studies including maternal nutrition during pregnancy and offspring neuroimaging.

	Study Design and Sample Size (*N*)	Maternal Nutrition Assessed	Nutrition Assessment Tool/Source	Nutrition Assessment Gestation	Neuro-Imaging Method	Offspring Age at Imaging	Main Outcomes	Citation
Food Categories	Prospective and longitudinal *N = 1936*	Food categories	Customized semi-quantitative food frequency questionnaire	10–14 w, 22–26 w, and 30–34 w	ultrasound	22–24 w, 30–32 w, and 34–36 w prenatal	Fetal head circumference (HC) was positively associated with a pattern of high proportion of more animal internal organs, thallophyte, and shellfish intake in the second trimester and more meat/fewer nuts intake in the third trimester, respectively. Fetal HC was negatively associated with a pattern of a high proportion of vegetables/fish intake and more snacks/fewer eggs consumed during the second trimester, respectively.	[25]
	Prospective and cross-sectional *N = 47*	Food and beverage categories	24 h food frequency recall survey	24 w	ultrasound	24 w prenatal	Seafood consumption frequency during pregnancy was positively associated with fetal biparietal diameter (BPD). Maternal soda consumption showed a negative relationship with fetal HC.	[26]
	Originally from a randomized clinical trial *N = 56*	Food categories	3-day food diaries, fish consuming frequency questionnaire	First, second, third trimester and fish frequency questionnaire only at third trimester	EEG	2.1 y	Fish consumption frequency in the third trimester was positively associated with higher pattern-reversal visual evoked potentials component amplitude.	[27]
	Prospective and longitudinal *N = 417*	Food categories	Simplified food questionnaire	24–28 w	ultrasound	27.8 w prenatal	Frequency of ultra-processed food consumption was negatively associated with fetal HC in late pregnancy.	[28]
Macro-nutrients	Prospective and longitudinal *N = 94*	Fatty acids	Fasting venous blood	13.1 w, 20.5 w, 30.5 w	MRI	25.3 d	Maternal saturated free fatty acids measured in fasted blood samples during pregnancy were inversely associated with neonatal hypothalamic microstructure characterized by mean diffusivity.	[29]
	Prospective and longitudinal *N = 321*	Fatty acids	Blood	8–13 w	ultrasound	10–40 w prenatal	Higher maternal plasma phospholipid even-chain and very long even-chain saturated fatty acids (SFAs) were inversely associated with fetal HC and BPD at early pregnancy. Odd-chain SFAs were positively associated with fetal HC and BPD in early pregnancy.	[30]
	Prospective and longitudinal *N = 6900*, *2354*	Fatty acids	Venous blood	20.6 w	ultrasound, MRI	20.6 w and 30.4 w prenatal, 9–11 y	Maternal plasma *trans* fatty acid concentration during mid-pregnancy was inversely associated with fetal HC in the third trimester and the growth of fetal HC from the second to the third trimester.	[31]
	Prospective and longitudinal *N = 1553*	Fatty acids	Non-fasting venous blood	Mid-gestation	MRI	9–11 y	Maternal omega-3 polyunsaturated fatty acids (PUFAs) (including long-chain and short-chain) concentration during mid-pregnancy positively correlated with children’s total gray matter/white matter volume.	[32]
	Randomized double-blind placebo-controlled trial *N = 86*	Fatty acids	Supplementation clinical trial	Early pregnancy	MRI	0–28 d	Docosahexaenoic acid (DHA) and eicosapentaenoic acid (EPA), together with arachidonic acid (ArA), were associated with larger total brain volume, total gray matter volume, and corpus callosum and cortical volumes in male neonates.	[33]
	Originally from a randomized clinical trial *N = 19*, *55*	Fatty acids	Fasting blood	Third trimester	EEG	2.1 y	Maternal monounsaturated fatty acid in serum phospholipid fatty acids at the third trimester of pregnancy showed no correlation with visual evoked pattern-reversal potentials (ERP). Maternal dietary intake of SFA during pregnancy was negatively correlated with EPR.	[27]
	Randomized controlled trial *N = 136*	Fatty acids	Supplementation clinical trial	20 w to delivery	EEG	8.5 y	Maternal omega-3 PUFAs supplementation was not associated with attention system indicated by ERP efficiency scores in a set of left-frontal-hemisphere channel.	[34]
	Randomized clinical trial and longitudinal *N = 74*	Fatty acids	Supplementation clinical trial	22 w to delivery	MRI	10 y	Maternal omega-3 PUFAs supplementation was not associated with total brain volume assessed.	[35]
	Follow-up of double-blind randomized clinical trial *N = 85*	Fatty acids	Supplementation clinical trial	20 w to delivery	MRI	9.5–10 y	Maternal omega-3 PUFAs supplementation was associated with weaker brain functional connectivity of children in the default mode, sensorimotor, and frontal-parietal networks.	[36]
	Prospective and longitudinal *N = 41*	Total/added sugar	24 h dietary recalls	Second trimester	MRI	0–21 d	Maternal total and added sugar consumption were inversely associated with infant brain mean diffusivity (MD) values throughout all of the cortical mantle, including the posterior periphery.	[37]
	Prospective and cross-sectional *N = 156*	Protein	24 h recall and food frequency questionnaire	Third trimester	ultrasound	16–38 w prenatal	Higher fetal HC and BPD were associated with maternal protein consumption of 50–70 g/day rather than <50 g/day or >70 g/day measured in the third trimester.	[38]
Micro-nutrients	Prospective and longitudinal *N = 2095*	Folate	Venous blood	13.3 w	Ultrasound and MRI	Late pregnancy, 6–8 y, 9–11 y	Maternal folate deficiency during pregnancy was associated with smaller total brain volume and smaller cerebral white matter in children aged 9–11 years. Children exposed to deficient folate concentrations in utero had persistently smaller brains compared to controls from the third trimester to childhood.	[39]
	Prospective and longitudinal *N = 256*	Folate	Venous blood	13.5 w	MRI	6–8 y	Low prenatal folate levels were associated with a smaller total brain volume.	[40]
	One retrospective cohort and two independent observational cohorts *N = 292*, *1078*	Folate	U.S. folate fortification rollout information	Any part of gestation	MRI	8–18 y	Lower maternal folate level was associated with lower cortical thickness in the bilateral frontal and temporal region and delayed cortical thinning in temporal and parietal regions.	[41]
	Prospective and longitudinal *N = 190*	Folate/Vitamin B12	Blood samples	18 w, 28 w	MRI	22.3 y	Mothers exposed to low vitamin B12 at mid-pregnancy simultaneously with higher folate resulted in less cortical thickness in multiple temporal and parietal areas of offspring. Higher maternal folate was associated with greater subcortical volume in the high vitamin B12 group but lower volume in the low vitamin B12 group of offspring.	[42]
	Randomized controlled trial *N = 136*	Folate	Supplementation clinical trial	20 w to delivery	EEG	8.5 y	Folate supplementation from the 20th week of pregnancy until delivery improved children’s ability to solve response conflicts reflected by higher activation of the mid-cingulate cortex.	[34]
	Randomized controlled trial *N = 68*	Folate	Supplementation clinical trial	14 w to delivery	MEG	11 y	Assessment of neuronal responses to a language task showed increased power at the Beta and High Gamma bands in children from folate-supplemented mothers, suggesting more efficient semantic processing of language.	[43]
	Randomized controlled trial *N = 132*	Vitamin B12	Supplementation clinical trial, venous blood	14 w gestation to 6 w postpartum	EEG	6 y	Maternal vitamin B12 supplementation during pregnancy did not make a difference in children assessed for attentional resource allocation and brain efficiency measures using ERP amplitudes and latencies. However, higher third-trimester maternal methylmalonic acid concentration was associated with lower ERP amplitude in children.	[44]
	Prospective and longitudinal *N = 149*	Choline	Non-fasting venous blood	16 w, 28 w	EEG	28 d	Lower maternal choline was associated with decreased offspring auditory P50 inhibition, a marker of inhibitory neuron development.	[45]
	Prospective and longitudinal *N = 162*	Choline	Serum	16 w	EEG	28 d, 90 d	Greater choline levels in mothers with infections were associated with improved newborn P50 inhibition of auditory cerebral response, mitigating the effect of infection.	[46]
	Randomized controlled trial *N = 93*	Choline	Supplementation clinical trial	17.2 w to delivery	EEG	33 d, 89 d	More choline-treated infants suppressed the P50 response, compared to placebo-treated infants at the fifth postnatal week. A CHRNA7 genotype associated with schizophrenia diminished P50 inhibition in the placebo-treated infants, but not in the choline-treated infants.	[47]
	Prospective and longitudinal *N = 2957*	Vitamin D	Venous blood/cord blood	18.1–24.9 w, 27.6–43.4 w, respectively	MRI	9–11 y	Children exposed to persistently deficient 25(OH)D concentration from mid-gestation to delivery showed less cerebral gray matter and white matter volumes, as well as smaller surface area and less gyrification at 10 years.	[48]
	Prospective and longitudinal *N = 90*	Iron	Venous blood	37–41 w	MRI	3–5 d	The severity of iron deficiency anemia during the third trimester was inversely associated with neonatal hippocampal volumes.	[49]
	Prospective and longitudinal *N = 40*	Iron	24 h dietary recall	34–36 w	MRI	19.6 d	Maternal iron intake during pregnancy was inversely associated with neonatal FA values predominately in cortical gray matter.	[50]
	Prospective and longitudinal *N = 1048*	Iron	Non-fasting venous blood	6–18 w	MRI	8–12 y	High maternal ferritin was associated with lower child intelligence quotient and smaller brain volume.	[51]
	Prospective and longitudinal *N = 337*	Iron	24 h recall	Mid-pregnancy	ultrasound	Mid-pregnancy	In the babies of mothers in the third tertile of iron intake (>17.04 mg), biparietal diameter, abdominal circumference, and femur length were lower, respectively, than the babies of mothers in the second tertile of iron intake (11.49 ~ 17.04 mg).	[52]
	Prospective and longitudinal *N = 990*	Iodine	Urinary iodine concentration and creatinine	<18 w, 18–25 w	MRI	10 y	Results suggest a curvilinear association between maternal UI/Creatine and children’s brain volume.	[53]
	Prospective and longitudinal *N = 41*	Zinc	24 h dietary recalls	First, second, third trimester	MRI	21 d	Maternal zinc intake inversely associated with infant FA toward the midbrain in each trimester and with infant MD in the peripheral cortex during the first and third trimesters. Maternal zinc also correlated positively with infant rCBF toward the hindbrain.	[54]
	Prospective and longitudinal *N = 44*	Sodium	3-day food records	First, second, third trimester	MRI	14 d	Maternal sodium intake during the first trimester of pregnancy was negatively associated with neonatal FA, involving the anterior corona radiate, parietal white matter, posterior limb of internal capsule, external capsule, and temporal white matter.	[55]

## Data Availability

Not applicable.
